# Bartholin gland carcinoma: A case report

**DOI:** 10.3892/ol.2014.2200

**Published:** 2014-05-30

**Authors:** PING ZHAN, GANG LI, BIN LIU, XI-GUANG MAO

**Affiliations:** 1 Department of Gynaecology and Obstetrics, Affiliated Hospital of Luzhou Medical College, Luzhou, Sichuan 646000, P.R. China; 2 Department of Pediatrics, Affiliated Hospital of Luzhou Medical College, Luzhou, Sichuan 646000, P.R. China

**Keywords:** Bartholin gland carcinomas, five-year survival rate

## Abstract

Bartholin gland carcinomas (BGCs) are extremely rare tumors accounting for <1% of all female genital malignancies. The current study presents a 49-year-old female with an eight-year history of BGC. A mass was identified in the vulva and the patient underwent an excisional biopsy, which revealed a left Bartholin adenoid cystic carcinoma. The patient subsequently received surgery, chemotherapy and biological therapy, and has survived. Therefore, the present case indicates that surgery is important for the treatment of BGC, however; multimodal therapy may be a more effective treatment strategy.

## Introduction

Bartholin gland carcinomas (BGCs) are extremely rare tumors, accounting for <1% of all female genital malignancies. BGCs commonly occur in postmenopausal female with a median age of 50 years at presentation (1). The pathogenesis of BGC remains unknown; however, it may be associated with infections of the vulva and Bartholin glands. Previous studies have reported that BGC is associated with the etiologically of the human papillomavirus (HPV) infection, particularly with HPV type 16 (2,3). Due to the deep location of Bartholin glands and the lack of early clinical symptoms, BGC is commonly misdiagnosed. Bilateral primary BGC is extremely rare. Furthermore, BGC is classified into various histological types, including adenocarcinoma, squamous, adenoid cystic, keratosis gland, transitional cell and undifferentiated carcinomas, among which adenocarcinoma and squamous carcinoma are the most common (3). The major transfer pathways include local invasion, lymph node metastasis and hematogenous metastasis. The diagnosis of BGC is dependent on pathological analysis and BGC is predominantly treated by surgery, and integrated applications of chemotherapy and radiotherapy are adopted for mid-term or advanced cases. Although the traditional management of BGC is surgery, clinical studies have demonstrated that multimodal therapy may be a more effective strategy (4–6). The prognosis of BGC patients is poor due to delayed treatment that results from misdiagnosis. Therefore, the aim of the present study was demonstrate the importance of early diagnosis and treatment of BGC, thus, improving the prognosis of patients with BGCs.

## Case report

A 49-year-old postmenopausal female was referred to the Affiliated Hospital of Luzhou Medical College (Luzhou, China) with an eight-year history of BGC. Written informed consent was obtained from the participant. This study was conducted in accordance with the Declaration of Helsinki and with approval from the Ethics Committee of the Affiliated Hospital of Luzhou Medical College.

**Figure 1:**
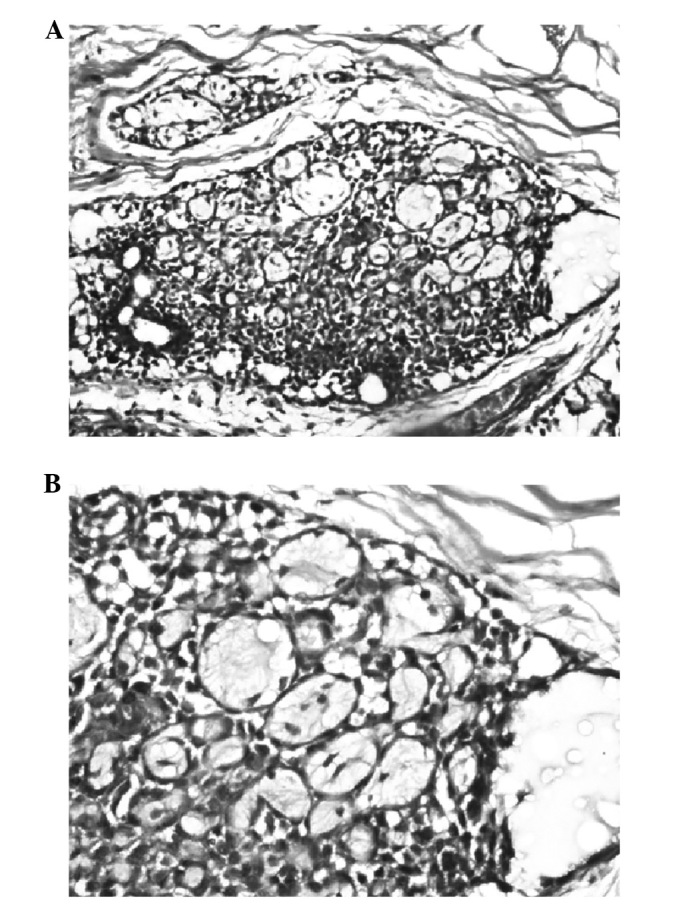
Left Bartholin adenoid cystic carcinoma. Hemotoxylin and eosin staining. Magnification; (A) ×200 and (B) ×400.

**Figure 2:**
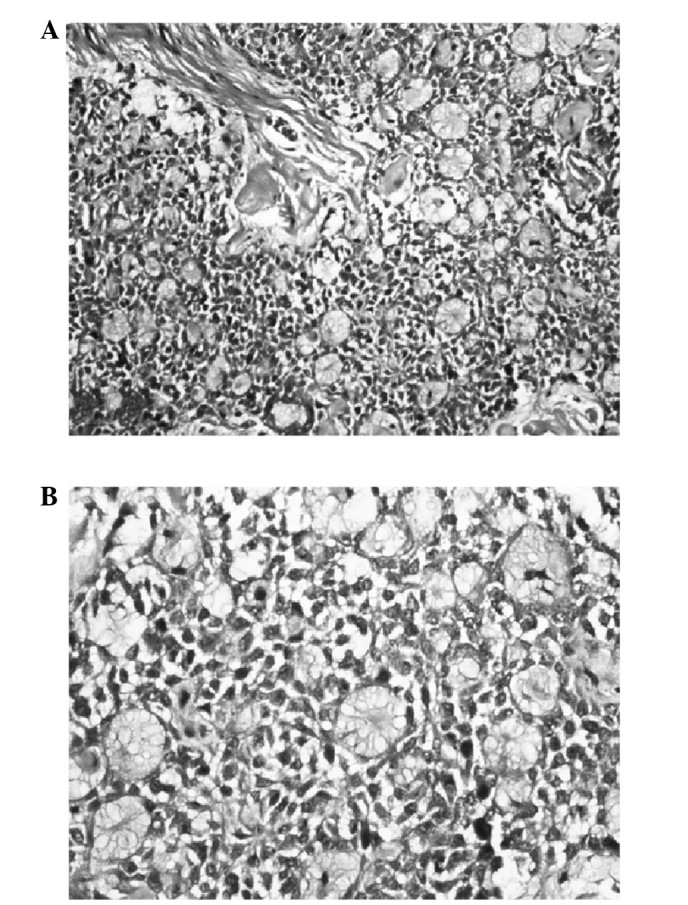
Left lower abdomen metastatic adenoid cystic carcinoma. Hemotoxylin and eosin staining. Magnification; (A) ×200 and (B) ×400.

Eight years ago, the patient presented with a hard, fixed, ill-defined nodule (measuring 3×3×2 cm^3^), which was identified unintentionally in the vulva. A left Bartholin cyst was diagnosed at the Luzhou Natural Gas Chemical Plant Worker Hospital (Luzhou, China) and the patient underwent left Bartholin cyst fenestration. After two years, the patient presented to the Affiliated Hospital of Luzhou Medical College with a new mass in the same location and underwent an excisional biopsy. The biopsy specimen was sent to the Department of Pathology, Affiliated Hospital of Luzhou Medical College, and pathologically diagnosed as left Bartholin adenoid cystic carcinoma (ACC). The patient subsequently underwent a bilateral inguinal lymphadenectomy in the Department of Gynaecology and Obstetrics, Affiliated Hospital of Luzhou Medical College to cure the vulvar cancer. The pathological examinations were consistent with the previous examination and revealed that the lymph node exhibited reactive hyperplasia; however, no cancer cells were found (Fig. 1). Postoperative magnetic resonance imaging of the pelvis did not indicate any disease. The patient received local radiation therapy (dose, 5,000 cGY/25F/5W) and immunotherapy. Notably, three years subsequently, a palpable mass measuring 1×1×1 cm^3^ was identified at the right side of the perineum and cancer cells were detected via cytology. The patient underwent simple mass excision. Pathological analysis revealed an ACC of Bartholin’s gland. Chemotherapy and biological therapy were performed. Positron emission tomography-computed tomography revealed small nodules in the left upper lobe and lower left abdominal subcutaneous nodules with increased glucose metabolism after six months. An abdominal wall mass excision biopsy showed left lower abdominal metastatic ACC four months later (Fig. 2). The patient underwent four cycles of paclitaxel, cisplatin and fluorouracil chemotherapy; however, chemotherapy was terminated as a result of severe side-effects. The patient then underwent six cycles of biotherapy at intervals of two to 12 months. Currently, and more than five years after initial diagnosis, the patient continues to survive.

## Discussion

According to the literature, >300 cases of BGC have been reported. The major histological types of BGC include squamous cell and adenocarcinoma, and the other types include adenoid-cystic, transitional or undifferentiated carcinomas; ACC accounts for ~25% of all BGCs (6). An extensive search of the currently available cases revealed that only 62 were ACC of Bartholin’s gland (7). BGCs are slow-growing tumors associated with frequent recurrences that exhibit local invasion, and metastasis to tissues and/or organs. Bones and the lungs are the most common sites of distant recurrence. Unilateral BGC is common, while bilateral BGC is extremely rare. In the present study, the patient was diagnosed with, and treated for, left Bartholin ACC. However, three years later, right Bartholin ACC was diagnosed with metastases to the abdominal wall and lungs. As a pathological analysis was not initially performed, whether the right Bartholin ACC was a primary cancer or metastatic carcinoma remains unknown. This locally aggressive malignant neoplasm is commonly found in postmenopausal females and occurs occasionally in women aged <40 years (8). Treatment following initial recurrence extends the survival of patients, and overall survival may reach 75 and 58% at five and 10 years, respectively (3). As a result of numerous treatment modalities, the patient in the present case has survived for more than five years after the initial diagnosis of BGC.

BGCs are treated with various modalities, either alone or in combination, including surgery, chemotherapy and radiotherapy. Due to the lack of previous cases, no consensus regarding the optimal treatment of BGC has been established.

The majority of authors recommend surgery as the primary treatment when tumor invasion is limited. Major surgical treatment methods include radical vulvectomy and inguinal and pelvic lymph node dissection. However, the benefits of performing either unilateral or bilateral inguinal-femoral lymphadenectomy remain controversial. Leuchter *et al* (1) support bilateral dissection and demonstrated that the inguinal-femoral lymph node status markedly affects survival. Copeland *et al* (9) indicated that unilateral dissection and postoperative adjuvant radiation are adequate to treat negative nodes in the clinical setting. In the present study, bilateral dissection was the preferred treatment method. In addition, our patient was treated with adjuvant postoperative radiation, chemotherapy, immunotherapy and biological therapy. However, the benefits of performing aggressive surgical procedures alone and the advantages of surgery compared with other treatment modalities for BGC were not investigated. A wide range of surgical procedures are available, however, surgery is extensive and often the incisions do not heal well, which severly affect patient’s quality of life.

Radiotherapy is widely used for the treatment of tumors, and the benefits of radiotherapy in the treatment of BGC are significant. López-Varela *et al* (6) found that overall survival with primary radiation or chemoradiation therapy was similar to the surgical series reported by Leuchter *et al* (1) (71%) and Cardosi *et al* (67%) (5), while it was inferior to Copeland *et al* (84%) (9). This phenomenon may be associated with the age and clinical stage of the populations. The aim of primary radiation therapy is to achieve the same outcome as surgery, reduce morbidity and optimally preserve normal function (6). Postoperative adjuvant radiotherapy has been shown to be effective in controlling ACC in patients with positive margins and local recurrences (10). However, certain short-term secondary effects of radiation therapy remain, including moist desquamation, erythema/dermatitis, bowel symptoms (including diarrhea, constipation and temporary incontinence) and dry desquamation (6). Fortunately, these symptoms are easily cured.

Chemotherapeutic agents, such as cisplatin and 5-fluorouracil are commonly used to treat BGC in combination with radiation therapy in clinics. Various cytostatic agents have been assessed, however, the results were poor (11). Chemotherapeutic agents enhance the effects of radiation via radiosensitization and direct cytotoxicity; however, chemoradiation destroys the tumor and the gland. Previous studies have achieved marked effects by treating advanced cancers of the vulva with radiation and sensitizing chemotherapy (12,13). Although surgery appears to be superior to radiation alone in eliminating occult metastases among vulvar cancer patients without clinically apparent metastasis (14); chemoradiation may sterilize occult metastases near the primary site, thus reducing the requirement for wide resection margins.

Currently, the optimum treatment method for BGC has not been determined, and the efficacy of radiation and chemotherapy has not been clearly established. However, certain investigators hypothesize that radiation therapy or chemoradiation offer effective alternative strategies to surgery for the treatment of BGC, whilst preserving genital function and maintaining low levels of morbidity (4,6). In addition, early diagnosis combined with a radical vulvectomy and bilateral inguinal femoral lymph node dissection may optimize the patient’s likelihood of survival (10). Patients that do not exhibit metastatic lesions at early diagnosis should undergo cancer lesion exeresis to reduce the tumor payload and increase the efficacy of radiotherapy. When the cancer lesion is removed during surgery, cancer cells may fall off and result in implantation metastasis. In conclusion, multimodality therapy may be a particularly effective treatment option, as indicated by the present study. However, by increasing the number of reported cases of BGC, the optimal treatment strategy for this type of carcinoma may be determined.
